# Case Report: The Different Fates of Three Aneurysms: Diagnosis and Treatment Strategies for Unruptured Intracranial Aneurysms With Other Intracranial Diseases

**DOI:** 10.3389/fsurg.2022.863718

**Published:** 2022-05-10

**Authors:** Gaochao Guo, Liming Zhao, Ruiyu Wu, Bingqian Xue, Shao Zhang, Hao Liang, Tao Gao, Yuxue Sun, Yang Liu, Chaoyue Li

**Affiliations:** ^1^Henan Provincial People's Hospital, Cerebrovascular Disease Hospital, Zhengzhou, China; ^2^Department of Neurosurgery, Zhengzhou University People's Hospital, Zhengzhou, China; ^3^Department of Neurosurgery, People's Hospital of Henan University, Zhengzhou, China

**Keywords:** aneurysm, unruptured intracranial aneurysm (UIA), intracranial diseases, surgery, management strategy

## Abstract

Intracranial aneurysms are vascular diseases characterized by local aneurysms of intracranial arteries. Their etiology involves a variety of environmental and genetic factors. Unruptured intracranial aneurysms (UIAs) are more common in intracranial aneurysms, but once an aneurysm is ruptured, the fatality rate and disability rate are extremely high. Therefore, accurate assessment of each step in the detection of intracranial aneurysms, assessment of the risk of rupture, formulation of treatment strategies, and treatment benefits will help to better treat the disease. At present, the treatment of intracranial aneurysms is limited. Except for surgical intervention, there are no other effective methods. Therefore, when a patient has a UIA, the formulation of treatment and management strategies is a difficult issue facing neurosurgery. This article introduces the choice of different treatment strategies for 3 patients with intracranial aneurysms complicated with other diseases and reviews the literature.

## Introduction

A ruptured intracranial aneurysm can cause fatal subarachnoid hemorrhage (SAH), a common critical condition in neurosurgery (1). The peak age of onset is generally between 50 and 60 years old (2, 3). The current cure for this type of aneurysm is only endovascular treatment and craniotomy to clamp the aneurysm (4, 5). Unruptured intracranial aneurysms (UIAs) are the most common. A multinational, multicenter study found that the incidence of UIAs was as high as 3.2% (3). However, with the continuous improvement of head imaging technology and the wide application of high-field MRI and magnetic resonance angiography (MRA), the detection rate of UIA is still increasing; a cross-sectional study in China reported that the prevalence of UIA detected by MRA was as high as 7% (6, 7). The prevalence rate reached the highest point in patients who were between 55 and 64 years old, and the prevalence rate in women was significantly higher than that in men (8). A meta-analysis study involving the follow-up results of 3,907 people showed that the overall annual rupture rate of UIAs was ~1.9% (9, 10), while the results of the first phase of the International Unruptured Intracranial Aneurysm Study retrospective study showed that the annual UIA rupture rate was 0.95% (11). A prospective study published in China in 2021 pointed out that the annual rupture rate of UIAs was 1.0% (12). The abovementioned research reports show that with the advancement of medical technology, the detection rate of UIAs is gradually increasing. Clinicians must consider the patient's age, life expectancy, estimated risk of rupture, and the level of anxiety caused by awareness of the aneurysm; thus, the formulation of a reasonable diagnosis, treatment, and management plan is an urgent problem faced by neurosurgeons.

This article introduces 3 cases of intracranial aneurysm combined with other intracranial diseases, combined with the patient's condition, clinical symptoms, and risk assessment to formulate treatment strategies and clinical technical points.

## Patient 1

**Figure 1:**
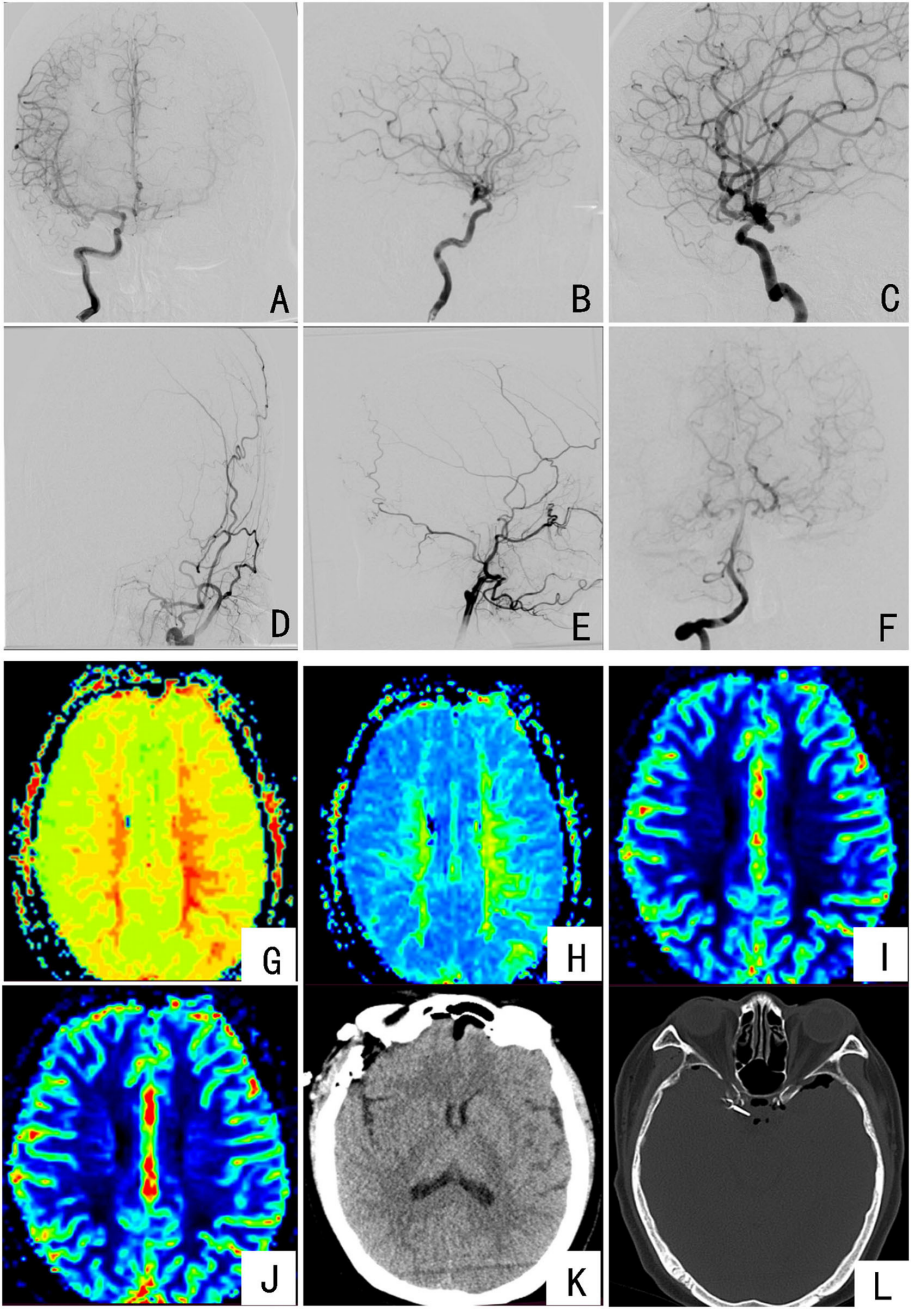
Preoperative and postoperative imaging examinations of the patient: **(A–C)** DSA imaging showing the right internal carotid artery communication segment considering an aneurysm; **(D,E)** DSA imaging showing intracranial occlusion of the left internal carotid artery; **(F)** DSA imaging showing the posterior circulation is clearly compensated to the left brain; **(G,H)** PWI indicates the left frontotemporal lobe TTP and MTT extension; **(I,J)** PWI indicates the CBV of the left frontal lobe increased and the CBV of the left temporal lobe decreased, but the bilateral CBF did not change significantly; **(K,L)** the results of the patient's head CT on the first day after surgery, with aneurysm clippings.

A 54-year-old female, was admitted to the fifth cerebrovascular ward of the Neurosurgery Department of Henan Provincial People's Hospital on 23 September 2021, mainly because the “physical examination found an intracranial aneurysm for more than 8 months.” More than 8 months before admission, the patient had a head injury and no positive signs and went to the local hospital for physical examination. MRA found that the right internal carotid artery traffic segment was considered an aneurysm; the left internal carotid artery (except part of the C7 segment) was severely stenotic or occluded. More than 2 months before admission, she underwent an MRA re-examination at the local hospital. MRA showed that the intracranial segment of the left internal carotid artery was not clearly visible, considering severe stenosis or occlusion; the right internal carotid artery traffic segment was considered an aneurysm; and there was a history of coronary heart disease for more than 7 months. After admission, the ambulatory blood pressure was improved (100–130 mmHg). Head digital subtraction angiography (DSA) showed a right internal carotid artery posterior communicating segment aneurysm and left internal carotid artery occlusion (Figures 1A–F). Head magnetic resonance perfusion weighted imaging (PWI) showed that the right basal ganglia may be an acute infarction, the left frontal lobe perfusion was delayed but relatively high perfusion, and the left temporal lobe perfusion was delayed and decreased (Figures 1G–J). There were no significant abnormalities in laboratory-related examinations. Neurological examination showed no positive signs. Combined with medical history and related examinations, the diagnosis was “right internal carotid aneurysm, left internal carotid artery occlusion, and coronary heart disease”.

According to the PWI staging of internal carotid artery occlusion, the left internal carotid artery occlusion is divided into Stage Ia, and Rinkel et al. (10) revealed that the overall annual rupture rate of UIAs in patients is 1.9%. After multidisciplinary discussion, one-stage aneurysm clipping was performed, and the left cerebral revascularization was performed in the second stage after 1 month. Key points for patient management during the perioperative period were as follows: Three days before surgery, infusion therapy was given to improve circulation, nourish cranial nerves, and protect brain function; due to the occlusion of the left internal carotid artery, the risk of perioperative cerebral ischemic stroke was higher, and the perioperative period strictly controlled the patient's non-invasive cuff pressure (arterial pressure 100–130 mmHg); intraoperative: during the beginning of anesthesia, surgical operation, and postoperative recovery, according to the patient's ambulatory blood pressure, the cuff arterial pressure was always maintained within the range of 100–130 mmHg to maintain the brain perfusion to prevent violent blood pressure fluctuations, causing cerebrovascular accidents; postoperative: head CT examination on the first day after surgery (Figures 1K,L); ECG monitoring for the first 3 days after surgery to maintain the cuff arterial pressure fluctuating in the range of 100–130 mmHg, 3,000–3,500 ml fluid rehydration volume; gradually reduced fluid volume on the fourth day after surgery, and discharged on the seventh day after surgery. Neurological examination at discharge was the same as before surgery.

## Patient 2

**Figure 2:**
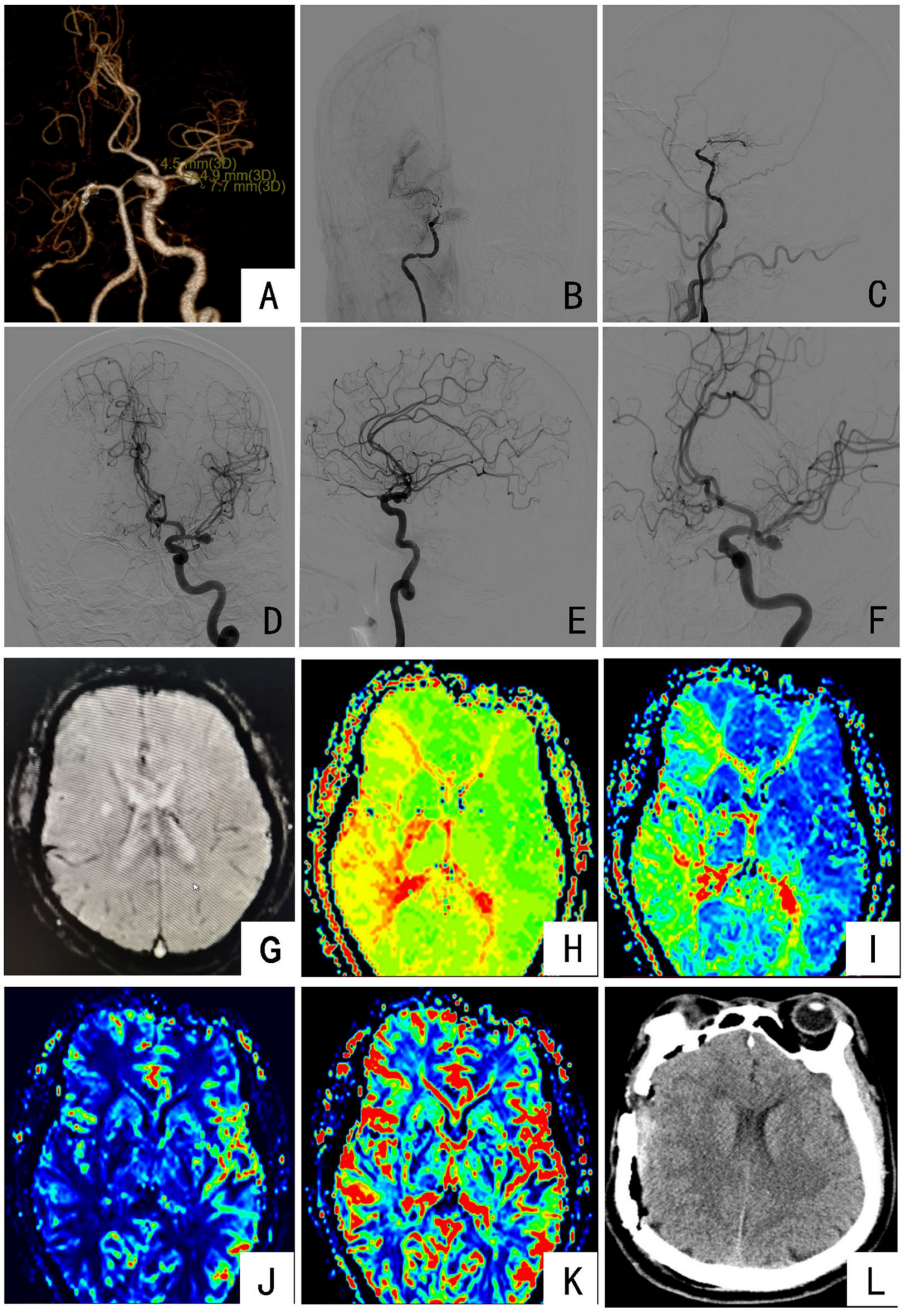
Preoperative and postoperative imaging examinations of the patient: **(A)** CTA showing occlusion of the right middle cerebral artery with the formation of small collateral vessels and the M1 segment of the left middle cerebral artery aneurysm; **(B,C)** DSA imaging showing that the cavernous sinus of the right internal carotid artery was occluded at the distal end, and the surrounding smoke-like blood vessels were formed; **(D–F)** DSA imaging showing that the end of the left middle cerebral artery M1 was an irregular aneurysm; **(G)** MRI indicates the right basal ganglia and corona demalaciasis; **(H,I)** PWI indicates bilateral oval center, radiated coronal area, temporoparietal occipital lobe, right frontal lobe and basal ganglia, and cerebellar hemisphere TTP and MTT prolongation; **(J,K)** PWI indicates a decrease in local CBF and no significant change in CBV; **(L)** the results of head CT on the first day after surgery.

A 68-year-old male, was admitted to the fifth cerebrovascular ward of the Neurosurgery Department of Henan Provincial People's Hospital on 24 September 2021, mainly due to “suddenly unfavorable left-hand limb activity and skewed mouth angle for 3 hours”. Three hours ago, the patient had a sudden left-side limb movement disorder and a skewed mouth angle, with no obvious positive signs. After 30 min of rest, the above symptoms disappeared; he then came to our hospital. There was a history of cerebral infarction in the past 15 days. After admission, the ambulatory blood pressure was improved (arterial pressure 130–150 mmHg); head and neck CT angiography (CTA) showed the following: the right internal carotid artery was slender, and the end was occluded; the right middle cerebral artery was occluded with small collateral vessels; the left middle cerebral artery M1 segment was tumor-like protrusion, possibly aneurysm (6.2 × 4.4 mm); the initial segment of the left internal carotid artery, the hard plaque of segment C6-7, and the lumen slightly narrowed (Figure 2A); head DSA showed the following: the right internal carotid artery cavernous sinus was distally occluded, and the left brain artery M1 end had an irregular aneurysm (Figures 2B–F); head MRI showed the right basal ganglia and corona infarction (Figure 2G); PWI showed a slight decrease in delayed perfusion in the bilateral hemispheres of the semicovale, radiographic coronal region, temporoparietal occipital lobe, right frontal lobe and basal ganglia region, and cerebellar hemisphere, suggesting hypoperfusion (Figures 2H–K). On admission to the hospital for physical examination, it's observed the consciousness, fluent speech, and execution as directed, normal high-level neurological function, left limb muscle strength V-, right limb muscle strength V, and normal muscle tone of limbs. Combined with the condition and related examinations, the diagnosis was “Transient ischemic attack (TIA), cerebral infarction, and left middle cerebral aneurysm”.

Combined with medical history and related examinations, the patient had cerebral ischemia as the main symptom. DSA examination found an unruptured aneurysm in the M1 segment of the left middle cerebral artery. After multidisciplinary discussion, one-stage right superficial temporal artery to middle cerebral artery (STA-MCA) anastomosis and encephalo-duro-arterio-myo-synangiosis (STA-MCA-EDMS) was performed, and the left-side aneurysm clipping operation was performed in the second stage after 1 month. Key points for patient management during the perioperative period were as follows: Three days before surgery, infusion therapy was given to improve circulation, nourish brain nerves, and protect brain function. Intraoperative: during the beginning of anesthesia, surgical operation, and postoperative recovery, it's always maintained the cuff according to the patient's dynamic blood pressure; arterial pressure should fluctuate in the range of 130–150 mmHg to maintain cerebral perfusion to prevent drastic fluctuations in blood pressure and cerebrovascular accidents. Postoperative: head CT examination on the first day after surgery (Figure 2L); ECG monitoring for the first 3 days after surgery to maintain the cuff arterial pressure fluctuating in the range of 130–150 mmHg. The fluid volume was gradually reduced on the fourth day after the operation, and the patient was discharged on the ninth day after surgery. Neurological examination at discharge was the same as before surgery.

## Patient 3

**Figure 3:**
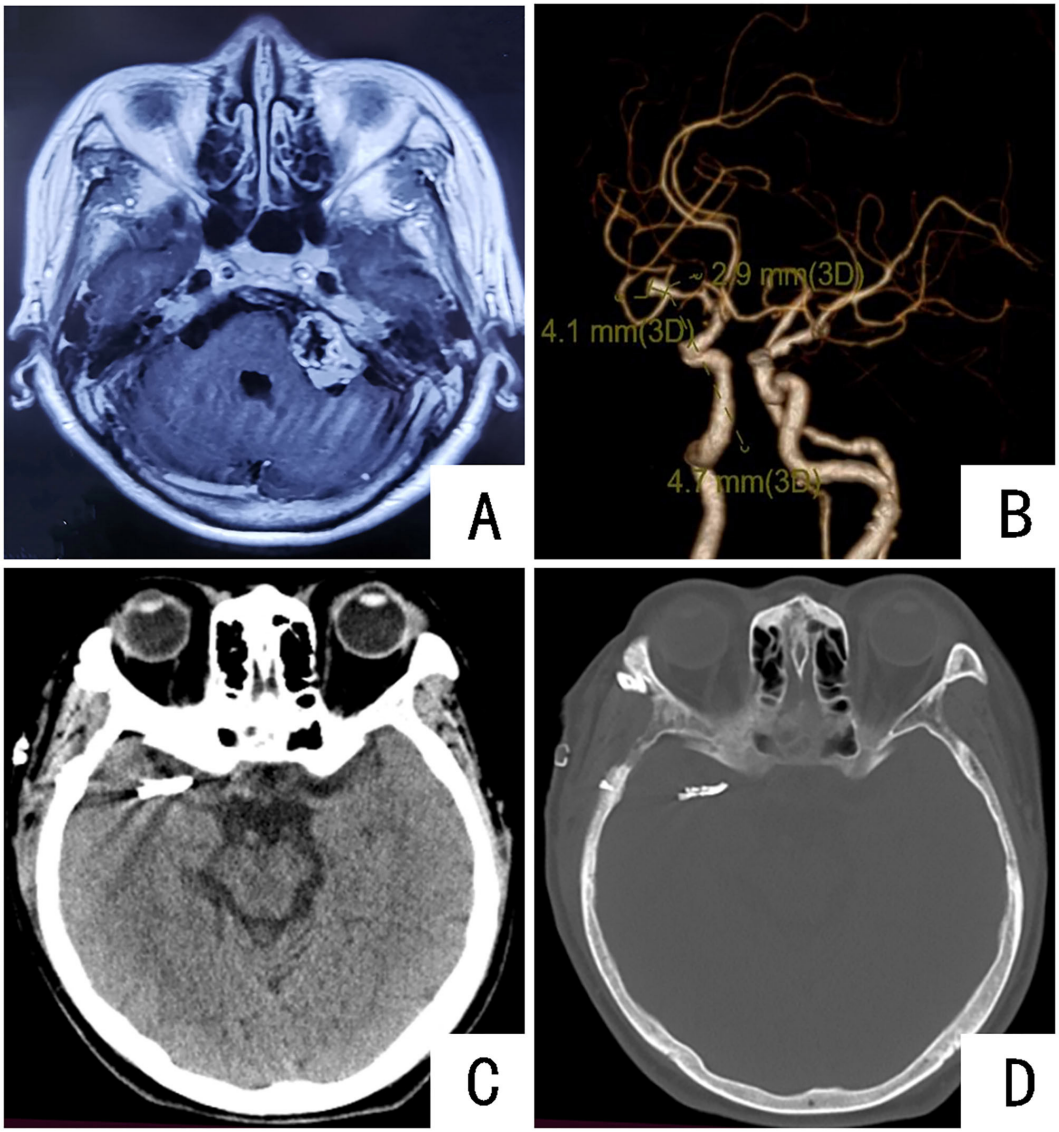
Preoperative and postoperative imaging examinations of the patient: **(A)** MRI showing space-occupying lesions in the left cerebellopontine area; **(B)** CTA showing the right middle cerebral artery M1 aneurysm; **(C,D)** CT results of the head of the patient on the first day after the operation. Aneurysm clippings can be seen.

A 60-year-old female, was admitted to the fifth cerebrovascular ward of the Neurosurgery Department of Henan Provincial People's Hospital on 27 September 2021, mainly due to “19 years of left ear hearing loss and intermittent dizziness for 11 days”. Nineteen years ago, the patient developed intermittent tinnitus with hearing loss and was not treated. Eleven days ago, due to dizziness, as visual rotation—without facial numbness, pain and cough—she visited a local hospital, and the head MRI showed the following: (1) the left cerebellopontine angle area occupying lesions, considering acoustic neuroma; (2) multiple luminal infarcts in the right lateral frontal lobe and the semi oval center of the double basal ganglia (Figure 3A). For further treatment, she then came to our hospital. She had a history of hypertension for more than 19 years, was untreated, and underwent total hysterectomy 5 years ago. After admission, head and neck CTA showed the following (Figure 3B): (1) The thickness of the right middle cerebral artery M1 segment was uneven, and local nodular protrusion was considered. A possible aneurysm was considered (4.8 × 2.9 mm). (2) The right anterior cerebral artery A2 segment had slender stenosis, the right lateral vertebral artery was slender, and the V4 segment of the left vertebral artery had multiple localized stenoses; Neurological examination showed no positive signs,except for hearing loss in the left ear. Combined with the condition and related examinations, the diagnosis was “occupying lesions in the left cerebellopontine angle area, right middle cerebral aneurysm, cerebral infarction, hypertension, posttotal hysterectomy, and multiple intracranial arterial stenosis”.

Combined with the medical history, the patient presented with hearing loss for 19 years and intermittent dizziness for 11 days. MRI found a space-occupying lesion in the left cerebellopontine area. Acoustic neuroma was considered; due to dizziness, the patient underwent CTA to find an aneurysm of the M1 segment of the right middle cerebral artery. Looking at the literature, in a PHASES (referring to 6 factors, namely, population, hypertension, age, aneurysm diameter, aneurysm location, and bleeding history of another aneurysm) study that included 6 cohort studies, the rupture rate was as high as 1.4% within 1 year, and the average rupture rate within 5 years was as high as 3.4% (13). After admission, the patient was given treatments to stop dizziness and control blood pressure. The symptom of dizziness was significantly improved, and the arterial pressure was controlled at fluctuations of 120–140 mmHg. After multidisciplinary discussion, the first stage of right middle cerebral aneurysm clipping was performed, and the second-stage occupying resection of the cerebellopontine angle on the left side was performed after 1 month. Key points for patient management during the perioperative period were as follows: Three days before surgery, infusion therapy was given to improve circulation, nourish brain nerves, and protect brain function; intraoperative: during the beginning of anesthesia, surgical operation, and postoperative recovery, the cuff should be maintained in conjunction with the blood pressure monitoring value; in the ward, arterial pressure fluctuates in the range of 120–140 mmHg to prevent violent blood pressure fluctuations and cerebrovascular accidents; postoperative: head CT examination on the first day after surgery (Figures 3C,D); ECG monitoring for the first 3 days after surgery, with 3,000–3,500 ml rehydration volume; fluid volume was gradually reduced on the fourth day after surgery, and the patient was discharged on the eighth day after surgery. Neurological examination at discharge was the same as before surgery.

## Discussion

Unruptured intracranial aneurysm is a pathologically limited expansion of the vessel wall of the intracranial artery, and there is a risk of rupture. Approximately 90% of spontaneous SAHs are caused by the rupture of intracranial aneurysms (1, 14); thus, reasonable treatment and management are important methods to prevent UIA rupture. The choice of surgical options for patients with other intracranial diseases and UIAs that require surgery is a challenge for neurosurgeons.

This article introduces 3 cases of intracranial unruptured aneurysms combined with other intracranial diseases: “right internal carotid artery aneurysm combined with left internal carotid artery occlusion”, “left middle cerebral artery M1 aneurysm combined with right internal carotid artery occlusion”, and “right middle cerebral artery M1 segment aneurysm combined with the left cerebellopontine angle area”. Based on the comprehensive evaluation of the 3 patients, a personalized treatment plan was developed. Physical examination of Patient 1 revealed an aneurysm in the right internal carotid artery traffic segment combined with left internal carotid artery occlusion. Combined with the patient's medical history and related examinations, DSA showed that the posterior circulation and the right internal carotid artery to the left cerebral hemisphere were compensated; PWI showed that the left frontal lobe perfusion was delayed but there was relatively high perfusion, and the left temporal lobe perfusion was delayed and decreased, and it was in the compensatory phase; the patient's ischemic symptoms were not obvious. Combined with literature reports and multidisciplinary discussions, a one-stage craniotomy for aneurysm clipping was performed. Patient 2 was admitted to the hospital mainly due to ischemic attack. CTA revealed that the right internal carotid artery was occluded, and the left middle cerebral artery M1 terminal had an irregular aneurysm (6.2 × 4.4 mm). PWI showed that the right frontal lobe and basal ganglia area perfusion was delayed and decreased. A prospective cohort study based on 1,087 cases of UIAs <7 mm in diameter showed that the annual rupture rate of UIAs was 1.0% (12). Regarding the main symptoms of patients with intracranial ischemia, the perioperative assessment of the risk of stroke was much higher than the risk of intracranial aneurysm rupture, so a stage of right revascularization was established. Patient 3 was admitted to the hospital with hearing loss and intermittent dizziness. MRI and MRA revealed a space-occupying lesion in the left cerebellopontine angle and an M1 aneurysm in the right middle cerebral artery. Combined with the medical history, the incidence of the cerebellopontine region was likely to occur, but the patient had a history of hypertension and poor blood pressure control, the annual risk of UIA rupture was as high as 1.4% (15, 16), and the patient's dizziness symptoms were well controlled after medication. After discussion, a one-stage craniotomy for aneurysm clipping was performed.

The detection rate of UIAs is increasing with the development of imaging. Therefore, the formulation of diagnosis and treatment strategies has gradually become a hot and difficult issue. The results of a phase I retrospective study of the International Study of Unruptured Intracranial Aneurysms (ISUIA) showed that the annual rupture rate of UIAs was 0.95% (95% CI: 0.79–1.15%) (11), but for symptomatic UIAs, the risk of rupture was 4.4 times that of asymptomatic aneurysms (95% CI: 2.8–6.8) (17). In a retrospective analysis of 280 cases of intracranial aneurysms, approximately 75% of ruptured aneurysms were <10 mm in diameter, and 26.1% of ruptured aneurysms were <5 mm in diameter (18). However, in the analysis of risk factors for aneurysm rupture, it was found that epidemiological risk factors play an important role. Among them, hypertension, smoking, previous bleeding, and family history have a clear role (5, 13, 19). When patients display the above risk factors, the aneurysm should be treated more actively. The three cases of aneurysms in this article were all detected from non-primary causes, and there were no clear symptoms caused by the aneurysm. They were of different sizes and shapes, were not on the same side as another lesion, and could not be treated at the same time in one phase. Therefore, more attention was given to individual and accurate clinical diagnosis and treatment decision-making.

The 3 cases of UIAs combined with other intracranial diseases reported in this article are not representative. They only introduce and share the treatment experience of the team. In the follow-up, more similar cases will be collected and summarized, and related research and long-term follow-up will be conducted, providing a clinical basis for the development of diagnosis and treatment plans for these patients.

## Data Availability Statement

The original contributions presented in the study are included in the article/supplementary material, further inquiries can be directed to the corresponding author/s.

## Ethics Statement

Written informed consent was obtained from the individual(s) for the publication of any potentially identifiable images or data included in this article.

## Author Contributions

GG and LZ were responsible for writing the article and sorting out the data. RW, BX, SZ, HL, YS, and TG collected and sorted out the data. CL and YL revised the article and data. All authors contributed to the article and approved the submitted version.

## Funding

This work was supported by the Scientific and Technological Projects in Henan Province (222102310046) and Leading Talent Training in Henan Province (YXKC2021004).

## Conflict of Interest

The authors declare that the research was conducted in the absence of any commercial or financial relationships that could be construed as a potential conflict of interest.

## Publisher's Note

All claims expressed in this article are solely those of the authors and do not necessarily represent those of their affiliated organizations, or those of the publisher, the editors and the reviewers. Any product that may be evaluated in this article, or claim that may be made by its manufacturer, is not guaranteed or endorsed by the publisher.
